# Evaluating the role of nursing interventions in enhanced recovery after surgery for minimally invasive spine surgery: a retrospective analysis

**DOI:** 10.3389/fsurg.2025.1519135

**Published:** 2025-02-19

**Authors:** Dan Zhang, Hongmei Ding, Caiping Shen, Yanyan Liu, Nan Jia

**Affiliations:** Guangdong Clinical Research Academy of Chinese Medicine, The First Affiliated Hospital of Guangzhou University of Chinese Medicine, Guangdong, Guangzhou, China

**Keywords:** enhanced recovery after surgery, minimally invasive spine surgery, spine surgery, pain management, nursing interventions

## Abstract

**Background:**

Enhanced Recovery After Surgery (ERAS) protocols have revolutionized postoperative care, particularly in minimally invasive spine surgery (MISS). This study aims to evaluate the role of nursing interventions in improving patient outcomes and reducing healthcare costs within this framework.

**Methods:**

This retrospective cohort study evaluated 150 patients undergoing MISS at The First Affiliated Hospital of Guangzhou University of Chinese Medicine from January 2017 to December 2021. Of these, 75 were assigned to the conventional group and 75 to the ERAS group. The study compared conventional nursing care with the ERAS protocol, assessing clinical outcomes and hospital expenses.

**Results:**

The analysis revealed that implementing targeted nursing interventions significantly decreased the length of hospital stay (LOS) in the ERAS group compared to the conventional group (3.2 days vs. 4 days; *p* *<* 0.001). Moreover, the multivariate analysis demonstrated that the patients in the the conventional group had significantly higher odds of prolonged length of stay (LOS) as compared to the ERAS group (OR: 5.114; 95% CI: 2.345–11.152, *p* < 0.001). Furthermore, postoperative drainage volumes were markedly lower in the ERAS group than in the conventional cohort (*p* < 0.001). Opioid consumption was also reduced, with only 24% of patients in the ERAS group requiring opioids, compared to 45.3% in the conventional care group (*p* *=* *0.01*). Additionally, the ERAS protocol resulted in lower overall hospital expenses, highlighting its cost-effectiveness in enhancing patient outcomes.

**Conclusion:**

The implementation of targeted nursing interventions within the ERAS protocol significantly improves patient outcomes in MISS. The ERAS group demonstrated shorter hospital stays, reduced postoperative drainage, and lower opioid requirements compared to the conventional care group. Additionally, the cost-effectiveness of the ERAS protocol highlights its potential to enhance overall healthcare efficiency.

## 1 Introduction

The conventional approach to spine surgery typically involves open surgery, which requires large incisions to provide direct access to anatomical structures. However, advancements in medical technology have enabled the use of minimally invasive spine surgery (MISS), offering a less disruptive alternative that achieves comparable therapeutic outcomes with fewer and smaller incisions (1). This approach minimizes damage to surrounding musculature, leading to faster recovery, decreased postoperative pain, and improved patient outcomes. MISS has become a vital treatment option for conditions such as lumbar disc herniation (LDH) and spinal fractures.

Effective perioperative nursing interventions are crucial for optimizing outcomes after MISS. However, standard nursing programs often have limited impact on key clinical outcomes, such as hospital stay and complication rates, failing to meet the specific needs of MISS patients. To address these gaps, the Enhanced Recovery After Surgery (ERAS) programs were introduced at the Hospital Universitario Rigshospitalet in Copenhagen, Denmark, in 2001, led by Professor Henrik Kehlet (2). The ERAS protocol is a multimodal approach that reduces physical and psychological trauma for surgical patients, improves outcomes, and accelerates recovery by integrating optimization measures with evidence-based perioperative management. Nurses are crucial to effectively executing ERAS, actively contributing as assessors, educators, caregivers, and coordinators within the multidisciplinary team (3, 4). A study by Li et al. (5) showed that a nurse-led ERAS program for elderly patients undergoing lung surgery enhanced recovery and improved outcomes in geriatric patients post-lung surgery. Nurses have demonstrated growing leadership within the ERAS program, actively contributing to developing and implementing ERAS pathways, highlighting their leadership capabilities, and reinforcing confidence in their ability to expand ERAS principles across diverse medical specialties and disciplines, including elective and emergency care settings (3–7). ERAS protocols have been widely applied in various surgical procedures, including emergency laparotomy (8), hepatobiliary surgery (9), lumbar fusion surgery (10), colorectal surgery (11), and gynecologic oncology (12), as outlined in the ERAS Society's guidelines (https://erassociety.org/guidelines/).

This study aims to investigate the impact of different nursing care protocols on post-operative outcomes in patients undergoing minimally invasive spine surgery. Specifically, it evaluates the effectiveness of ERAS-based nursing interventions in improving recovery, minimizing complications, and enhancing patient outcomes compared to conventional nursing care.

## 2 Methods

Patient records were retrospectively reviewed from the hospital's electronic medical database. To maintain procedural consistency, all surgeries were performed by a specialized team of surgeons with expertise in minimally invasive techniques. Nursing care for the ERAS group adhered to evidence-based protocols, emphasizing multimodal analgesia, early ambulation, and nutritional optimization to promote swift recovery (13). This retrospective cohort study analyzed 150 patients who underwent minimally invasive spine surgery at The First Affiliated Hospital of Guangzhou University of Chinese Medicine between January 2017 and December 2021. Participants were allocated into two groups based on the postoperative care protocol: 75 patients received conventional nursing care, while 75 were managed under ERAS protocol. Patients in the conventional nursing care group received standard postoperative care without the ERAS protocol, while patients in the ERAS group received nursing care based on the ERAS protocol. This approach incorporates evidence-based interventions to optimize recovery, minimize complications, and enhance overall patient outcomes following minimally invasive spine surgery.

### 2.1 Ethical considerations

Ethical approval for the study was granted by the Institutional Ethics Committee of The First Affiliated Hospital of Guangzhou University of Chinese Medicine (Approval No.: K-2021-136). All procedures were conducted in compliance with relevant institutional and national ethical guidelines. Informed consent was not required due to the retrospective nature of the study.

### 2.2 Inclusion and exclusion criteria

The inclusion criteria for this study comprised adults over 18 years of age who underwent single-level minimally invasive spine surgery for degenerative spinal conditions. Only patients who were hemodynamically stable pre-operatively were included. The exclusion criteria ruled out individuals who had undergone multilevel spinal surgeries or had a history of prior spinal surgery, spinal infections, neoplasms, or spinal deformities. Additionally, patients with severe comorbidities, such as uncontrolled diabetes, cognitive impairments, or those with incomplete clinical data were excluded from the study.

### 2.3 Postoperative numeric rating scale

The Postoperative Numeric Rating Scale (NRS) is a crucial instrument for assessing pain levels in patients after surgery (14). It enables patients to indicate their pain intensity on a scale ranging from 0 (no pain) to 10 (worst pain), thereby providing a consistent metric for documentation. This scale assists healthcare professionals in monitoring variations in pain over time and modifying pain management approaches as needed.

### 2.4 Nursing interventions and ERAS protocol implementation

The optimal approach to implementing fundamental changes in postoperative pain management for spine surgery is through the ERAS paradigm, widely recognized as the most effective framework (10). Evidence-based guidelines and best practices have been established to develop rational pain management protocols. ERAS pain management protocols emphasize a multidisciplinary approach throughout the surgical process, aiming to improve pain control and reduce reliance on opioids. One such protocol, developed by the Cleveland Clinic, is an exemplary model of an evidence-based, rational pain management strategy for spine surgery. An ERAS nurse provided detailed information about the preoperative and postoperative stages, home medications, and potential discharge scenarios. Nurses remained available for ongoing communication with patients after discharge. Patients were encouraged to access online information about their treatment and to register for hospital admission in advance to minimize waiting times. Nursing interventions were systematically applied across four key phases: preadmission, preoperative, intraoperative, and postoperative. Table 1 presents a comparative summary of the key differences between conventional care and the ERAS approach across various phases of care.

**Table 1:** Comparison of conventional care and ERAS protocols in spine surgery recovery.

Phase	Conventional care	ERAS care
Preadmission	Basic patient assessment and surgery eligibility check	Comprehensive assessment of medical history, psychosocial factors, and prehabilitation (physical activity, nutrition optimization)
Preoperative	Standard fasting guidelines and basic preoperative education	Thorough preparation, including fasting, medication management, risk assessment, and psychological support
Intraoperative	Routine monitoring and analgesic administration	ERAS-specific pain management, close collaboration with the surgical team, adherence to the protocol for anesthesia and analgesics
Postoperative	Standard pain management, limited mobilization, delayed nutritional support.	Multimodal analgesia with opioid-sparing strategies, early mobilization, early nutritional support, continuous education, and early home follow-up

#### 2.4.1 Preadmission phase

During the preadmission phase, nurses conducted comprehensive patient assessments, including evaluations of medical history and psychosocial factors, to determine eligibility for surgery. Patients who met the inclusion criteria were included in the study. Education regarding the surgical procedure and the ERAS protocol was provided, empowering patients to participate actively in their recovery. Prehabilitation strategies, such as promoting physical activity and optimizing nutrition, were also implemented to enhance the patients' preoperative status.

#### 2.4.2 Preoperative phase

In the preoperative phase, nurses ensured patients were adequately prepared for surgery, including adherence to fasting guidelines and proper medication management. Comprehensive risk assessments were conducted to identify potential complications, and nurses facilitated consultations with anesthesiology to establish an appropriate anesthesia plan. Psychological support was provided to address patient anxiety and encourage open communication regarding the surgical process.

#### 2.4.3 Intraoperative phase

During the intraoperative phase, nurses were crucial in monitoring patient vital signs and overall status throughout the surgical procedure. Close collaboration with the surgical team ensured that all ERAS protocols were followed and timely interventions were made to maintain patient stability. Nurses administered analgesics and anesthesia in accordance with ERAS guidelines to minimize postoperative pain.

#### 2.4.4 Postoperative phase

Postoperatively, nursing interventions focused on effective pain management through multimodal analgesia strategies aimed at reducing opioid consumption. Continuous vital signs and patient recovery were monitored to identify and address complications promptly. Nurses encouraged early mobilization, assisting patients in getting out of bed and walking as soon as feasible, in accordance with ERAS principles. Nutritional support was provided to facilitate early oral intake, and patient education was reinforced regarding postoperative care, including pain management, activity levels, and potential complications. For pain management, an opioid-sparing multimodal strategy was employed, prioritizing the use of tapentadol and ibuprofen in the recovery room. Hydromorphone was administered if pain remained inadequately controlled with these agents. Patient education emphasized proper analgesic use, focusing on minimizing opioid consumption. Early home follow-up was facilitated by an ERAS team nurse, who was accessible 24/7 via phone or a dedicated mobile application.

### 2.5 Outcome assessment

The primary outcomes assessed in this study included the length of hospital stay (LOS), the postoperative numeric rating scale (NRS), the 28-day readmission rate, the 28-day reoperation rate, and the occurrence of complications. Secondary outcomes examined the financial impact of ERAS protocols. Additionally, perioperative factors such as blood loss and surgical drainage were evaluated. Moreover, opioid consumption was assessed to provide a comprehensive analysis.

### 2.6 Standardization of nursing interventions in ERAS protocol

To ensure consistency across nursing staff and patient cases, several standardization measures were implemented:

(a) **Protocol Training**: A key component of standardizing care was ensuring that all nursing staff involved in the study underwent comprehensive training. This training focused not only on the technical aspects of the ERAS protocol but also on its underlying rationale. Nurses were educated on the specific interventions to be implemented at each stage of patient care, such as preoperative education, postoperative mobilization, and pain management strategies. The training was designed to enhance staff confidence in the protocol and ensure a uniform approach across the team.
(b) **Standardized Guidelines**: In addition to training, we developed and disseminated detailed, evidence-based nursing guidelines for implementing the ERAS protocol. These guidelines were carefully crafted to reflect current research and best practices, ensuring that nursing interventions were consistent and aligned with the ERAS principles. These guidelines were made accessible to all nursing staff, and regular updates were provided as new evidence emerged or modifications to the protocol were necessary.
(c) **Checklists and Documentation**: To further enhance consistency, structured checklists were created for each step of the ERAS protocol. These checklists served as a practical tool for nurses to ensure that all required interventions were carried out as planned. The checklists also reminded staff to monitor specific patient needs, such as nutritional intake, mobilization, and pain management. Furthermore, compliance with each intervention was documented in the patient's medical records, allowing for continuous monitoring and evaluation of adherence to the protocol.
(d) **Interdisciplinary Collaboration**: Successful implementation of the ERAS protocol required ongoing communication and collaboration among various healthcare providers. Regular interdisciplinary team meetings were held to assess protocol adherence, discuss patient progress, and identify deviations or challenges. These meetings provided a forum for nurses, physicians, dietitians, physiotherapists, and other specialists to collaborate on care decisions and problem-solve any barriers to successful protocol implementation. These team discussions ensured that any issues related to protocol adherence were quickly identified and addressed, promoting consistency in patient care.

### 2.7 Statistical analysis

Continuous variables were expressed as mean (standard deviation) and median (interquartile range, IQR), while categorical variables were presented as frequencies and percentages. For comparisons, independent sample *t*-tests or rank sum tests were utilized for continuous variables, and *χ*^2^ tests or Fisher's exact tests were applied for categorical variables. In this study prolonged length of stay (LOS) is defined as a hospital stay of 4 days or longer (≥4 days). The relationship between various factors and length of stay (LOS) was examined using multivariate analysis. Odds ratios (OR), and 95% confidence intervals (CI) were computed to assess how different factors influenced LOS. Statistical analyses were conducted using SPSS version 24.0, with a *p*-value of less than 0.05 deemed statistically significant.

## 3 Results

### 3.1 Baseline characteristics of study population

A total of 150 patients participated in the study, with 75 assigned to the ERAS group and 75 to the conventional care group. Both groups were comparable at baseline; no statistically significant differences were identified. The ERAS group had a median age of 46.0 years (IQR: 44–64), while the conventional group's median age was 55.0 years (IQR: 44.5–67.5) (*p* *=* 0.17). Median BMI values were 24.5 and 25.4 kg/m^2^, respectively (*p* *=* 0.082). Gender, radiological characteristics (herniated disc, stenosis, facet arthritis), and comorbidities (diabetes, hypertension, heart failure) showed no significant variation between groups (all *p* > 0.05). The L4/L5 level was the most frequently operated on, with similar distributions across surgical levels (*p* *=* 0.653). ASA grade classification was also consistent, with most patients categorized as ASA grade 2 (*p* *=* 0.532). Table 2 presents the baseline characteristics of the study population.

**Table 2:** Comparison of general information between the two groups of patients.

Variables	ERAS group (*n* = 75)	Conventional group (*n* = 75)	*p*-value
Age (years)	46.0 (44–64.0)	55.0 (44.5–67.5)	0.17
Gender (Male)	34 (45.3)	40 (53.3)	0.327
BMI (kg/m^2^)	24.5 (23.5–25.5)	25.4 (23.9–25.5)	0.082
Radiological features			
Herniated lumbar disc (yes)	63 (84)	55 (73.3)	0.111
Stenosis of lumbar spine (yes)	47 (62.7)	48 (64)	0.865
Arthritis of the Lumbar Facet	41 (54.7)	32 (42.7)	0.142
Operation level			0.653
L3/L4	7 (9.3)	6 (8)	
L4/L5	50 (66.7)	46 (61.3)	
L5/S1	18 (24)	23 (30.7)	
Comorbidities			
Diabetes mellitus	25 (33.3)	15 (20)	0.065
Hypertension	28 (37.3)	30 (40)	0.737
Congestive heart failure	43 (57.3)	34 (45.3)	0.142
Cerebrovascular disease	5 (6.7)	9 (12)	0.262
Renal disease	17 (22.7)	12 (16)	0.301
ASA grade			0.532
ASA 1	9 (12)	5 (5.7)	
ASA 2	47 (62.7)	50 (66.7)	
ASA 3	19 (25.3)	20 (26.7)	

Values are expressed as the median (IQR) or *n* (%). ASA, American Society of Anesthesiologists class.

### 3.2 Outcome measures

The clinical outcomes of the ERAS and conventional care groups are summarized in Table 3.

**Table 3:** Comparison of clinical and economic outcomes between ERAS and conventional care groups.

Variables	ERAS group (*n* = 75)	Convention group (*n* = 75)	*p*-value
Length of hospital stay (days)	3.2 (2.7–4)	4 (3.1–5.3)	<0.001
Intraoperative hemorrhage (ml)	47 (43–54)	49 (45–55)	0.109
Post-surgical drainage volume (ml)	20 (17–23.5)	25 (20–27.5)	<0.001
Aggregate hospital expenses (CNY)	20,540 (18,970–21,850)	23,789 (21,725–24,945)	<0.001
Opioid consumption	18 (24%)	34 (45.3%)	0.01
Complications			
Incidence of durotomy	6 (8.0)	4 (5.3)	0.513
Infection at the surgical site	5 (6.7)	4 (5.3)	731
Incidence of epidural hematoma	2 (2.7)	2 (2.7)	0.999
Postoperative pain scores (NRS)			
Postoperative day 0	2 (1–3)	3 (2–4)	<0.001
Postoperative day1	2 (1–3)	3 (2–4)	<0.001
Postoperative day 2	2 (1–2)	2 (1–3)	0.12
Readmission within 28 days	3 (4)	4 (5.3)	0.699
Reoperation within 28 days	3 (4)	2 (2.7)	0.649

Values are expressed as the median (IQR) or *n* (%).

#### 3.2.1 Primary outcomes

**Figure 1:**
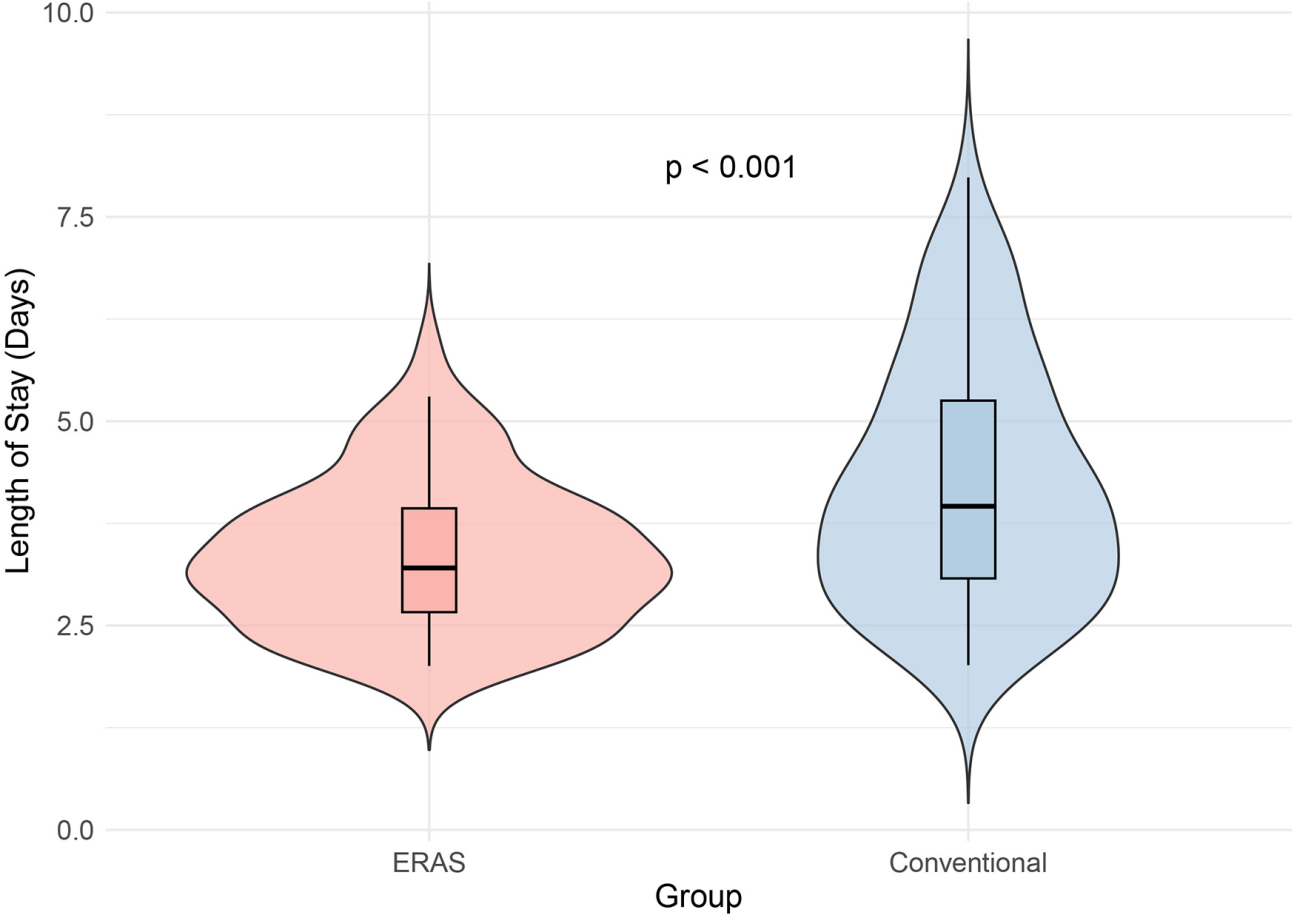
Violin plot illustrating length of stay in ERAS and conventional groups. The ERAS group had a significantly shorter hospital stay compared to the conventional group (*p* < 0.001), demonstrating the positive impact of ERAS protocols in reducing recovery time and hospital stay duration. This suggests that ERAS protocols can provide better outcomes in terms of faster recovery and discharge.

The ERAS group demonstrated a significantly shorter hospital stay compared to the conventional group (3.2 vs. 4 days, *p* < 0.001) (Figure 1; Table 3). Although intraoperative bleeding was similar between groups (47 vs. 49 ml, *p* = 0.109), the ERAS group had significantly lower post-surgical drainage volumes (20 vs. 25 ml, *p* < 0.001) (Table 3). Complication rates, including durotomy, surgical site infections, and epidural hematoma, were comparable between groups (all *p* > 0.05). Postoperative pain scores were significantly lower in the ERAS group on days 0 and 1 (both *p* < 0.001) but showed no difference on day 2 (*p* = 0.12). Readmission and reoperation rates within 28 days were also similar between the two groups (both *p* > 0.05) (Table 3).

#### 3.2.2 Secondary outcomes

The secondary outcomes focused on the financial impact of the ERAS protocols alongside an evaluation of perioperative factors, including blood loss, surgical drainage, and opioid consumption, as detailed in Table 3. The analysis revealed that implementing ERAS protocols significantly reduced aggregate hospital expenses in the ERAS group compared to the conventional group (20,540 vs. 23,789 CNY, *p* < 0.001). Regarding perioperative factors, intraoperative bleeding was similar between the groups (47 ml in the ERAS group vs. 49 ml in the conventional group, *p* = 0.109). However, the ERAS group exhibited a significantly lower volume of postoperative drainage (20 ml vs. 25 ml, *p* < 0.001). Opioid consumption was also reduced in the ERAS group, with only 24% of patients requiring opioids compared to 45.3% in the conventional group (*p* = 0.01). These findings highlight the economic benefits and improved perioperative management associated with ERAS protocols.

### 3.3 Multivariate logistic regression analysis

The multivariate logistic regression analysis for predictors of length of hospital stay (LOS) is summarized in Table 4. The results indicate that the type of care group (ERAS vs. Conventional) was a significant predictor of LOS. Patients in the conventional care group had significantly higher odds of prolonged LOS compared to those in the ERAS group, with an odds ratio (OR) of 5.114 (95% CI: 2.345–11.152, *p* < 0.001). This finding underscores the effectiveness of the ERAS protocol in reducing hospital stay duration. Other variables, including age (OR: 1.002; 95% CI: 0.972–1.032, *p* = 0.920), gender (male: OR: 0.863; 95% CI: 0.416–1.792, *p* = 0.693), and BMI (OR: 1.049; 95% CI: 0.823–1.336, *p* = 0.699), were not significantly associated with LOS. Among comorbidities, none showed a significant relationship with LOS. Cerebrovascular disease (OR: 2.657; 95% CI: 0.734–9.614, *p* = 0.136) had higher odds but lacked statistical significance. Congestive heart failure, renal disease, hypertension, diabetes, and lumbar conditions (disc hernia, spinal stenosis, arthritis) also showed no significant associations (*p* > 0.05). These findings suggest that comorbidities and demographic factors had limited impact on LOS.

**Table 4:** Multivariate logistic regression results for length of hospital stay (LOS) in ERAS vs. Conventional Groups.

Variables	OR (95% CI)	*P* value
Age	1.002 (0.972–1.032)	0.920
Gender (Male)	0.863 (0.416–1.792)	0.693
Congestive heart failure (Yes)	1.341 (0.619–2.905)	0.458
Cerebrovascular disease (Yes)	2.657 (0.734–9.614)	0.136
Renal disease (Yes)	0.912 (0.313–2.659)	0.866
Hypertension (Yes)	1.421 (0.626–3.223)	0.401
Diabetes (Yes)	0.922 (0.388–2.195)	0.855
Opoid use (Yes)	1.085 (0.503–2.340)	0.836
BMI	1.049 (0.823–1.336)	0.699
Lumbar disc hernia (Yes)	1.620 (0.629–4.174)	0.318
Lumbar spinal stenosis (Yes)	0.722 (0.337–1.547)	0.403
Lumbar arthritis (Yes)	1.029 (0.483–2.194)	0.940
Group (Conventional)	5.114 (2.345–11.152)	<0.001

Variable included in multivariate analysis: age, gender, congestive Heart failure, Cerebrovascular disease, renal disease, hypertension, diabetes, opoid use, BMI, lumbar disc hernia, lumbar spinal stenosis, lumbar arthritis, Groups (Conventional vs. ERAS).

## 4 Discussion

By implementing multimodal approaches, ERAS protocols have substantially improved patient outcomes in various surgical fields. These protocols have reduced complications, improved patient experiences, and shorter hospital stays. The primary findings of this study indicate that the implementation of the ERAS protocol led to a significant reduction in hospital length of stay (LOS) for patients undergoing MISS, decreasing from 4 to 3.2 days (*p* < 0.001). The multivariate analysis revealed that patients in the the conventional group had significantly higher odds of prolonged length of stay (LOS) as compared to the ERAS group (OR: 5.114; 95% CI: 2.345–11.152, *p* < 0.001). This finding supports the effectiveness of ERAS protocols in optimizing recovery and reducing hospital stays. The reduced LOS in the ERAS group may reflect improved post-surgical recovery, better management of complications, and a more efficient care pathway, all of which are key components of ERAS strategies. This result highlights the potential for ERAS protocols to improve clinical outcomes and hospital resource utilization. Additionally, the ERAS protocol was associated with improved postoperative outcomes, including lower pain scores on the numeric rating scale (NRS) and a marked decrease in opioid consumption, enhancing overall patient recovery. Furthermore, the analysis demonstrated a substantial financial impact, with aggregate hospital expenses notably lower in the ERAS group than in conventional care. These results underscore the effectiveness of the ERAS protocol in enhancing recovery while simultaneously reducing hospital costs for spinal surgery patients. Nursing interventions are critical to overcoming the challenges associated with implementing ERAS pathways. By addressing healthcare resources, influencing local medical policies, and reshaping traditional patient care concepts, nurses can facilitate the transition to ERAS models that promote efficient recovery and shorter hospital stays. Emphasizing the role of nurses in ERAS implementation can enhance the overall effectiveness of surgical care, ultimately improving patient outcomes and satisfaction.

This systematic review and meta-analysis (15) of 40 studies involving 7,885 patients evaluated the impact of ERAS protocols in gynecological surgery. The findings indicate that ERAS programs significantly improve outcomes by reducing hospital stays by 1.22 days, lowering readmission rates by 20%, and decreasing the incidence of ileus by 47%. These results highlight the effectiveness of ERAS in enhancing recovery and optimizing care in both benign and oncological gynecological surgeries. Noba et al. (16) investigated the clinical benefits, cost-effectiveness, and compliance with ERAS protocols in liver surgery and revealed that the implementation of ERAS protocols significantly reduced the length of hospital stay (LOS) by an average of 2.22 days (MD = −2.22; *p* < 0.001) and decreased the incidence of complications (RR = 0.71; *p* < 0.001). Additionally, hospital costs were notably lower in the ERAS group (SMD = −0.98; *p* < 0.01). These results confirm that ERAS protocols are safe and effective in hepatectomies, warranting further investigation into compliance and clinical outcomes.

Consistent with our findings, a meta-analysis (17) evaluating the effectiveness of ERAS protocols in improving postoperative outcomes for patients undergoing total hip arthroplasty (THA) and total knee arthroplasty (TKA) demonstrated a significant reduction in postoperative length of stay (LOS) in the ERAS group compared to the non-ERAS group (*p* *<* *0.01*). Additionally, the analysis reported a lower incidence of complications in the ERAS group (*p* *=* *0.03*). However, no significant difference was observed in the 30-day readmission rates (*p* *=* *0.18*). These findings underscore the potential of ERAS protocols to enhance recovery following THA and TKA, mainly by reducing LOS and complication rates. Similarly, a retrospective study (18) evaluated the safety, feasibility, and efficacy of an Enhanced Recovery After Surgery (ERAS) program in patients over 70 undergoing lumbar arthrodesis compared to traditional non-ERAS care. The results indicated a significantly reduced length of stay in the ERAS group (13.6 ± 4.0 days vs. 15.6 ± 3.9 days; *p* = 0.034) and a lower incidence of complications (8.3% vs. 20.9%; *p* = 0.048). Additionally, postoperative pain scores were notably lower on days 1 and 2 in the ERAS group, reflecting effective pain management strategies. With a compliance rate of 94%, this study provides additional evidence for the safety and effectiveness of ERAS protocols, especially in improving recovery outcomes for older adults undergoing lumbar spine surgery.

Further supporting these findings, another study (19) evaluated the impact of ERAS protocols on postoperative outcomes in spine surgery. The analysis included 386 patients, with 193 receiving spinal surgery following the ERAS protocol. The study reported a significantly reduced mean length of stay (LOS) in the ERAS group, averaging 2.6 days compared to 4.4 days in the control group (*p* < 0.001). Notably, the two groups had no significant differences in complications, readmission rates, postoperative pain, function, or satisfaction. These results further underscore the effectiveness of the ERAS protocol in reducing LOS without increasing adverse events among spinal surgery patients.

Furthermore, Webb et al. (20) evaluated the effects of the ERAS program on patients undergoing cytoreductive surgery (CRS) and hyperthermic intraperitoneal chemotherapy (HIPEC). Among 130 procedures analyzed, the mean length of stay decreased significantly from 10.3 days to 6.9 days (*p* = 0.007), and grade III/IV complication rates fell from 24% to 15%. Additionally, the ERAS group demonstrated a reduction in intravenous fluid requirements and opioid use, indicating improved postoperative recovery without increasing readmission rates or the incidence of acute kidney injury. This study supports the notion that ERAS protocols can enhance recovery experiences for patients undergoing complex surgical procedures, emphasizing the advantages of implementing evidence-based interventions in surgical care.

Moreover, our study identified significant differences in length of stay (LOS) compared to the study by Dragun et al. (21). While their research did not find a substantial reduction in hospital or ICU LOS with ERAS, our study demonstrated a notable decrease in hospital stay for the ERAS group. These differences in findings highlight the variability in outcomes associated with ERAS protocols, suggesting that institutional practices, patient demographics, and specific surgical procedures may influence the effectiveness of these protocols. For example, variations in preoperative counseling, anesthesia techniques, and postoperative care pathways can lead to discrepancies in length of stay and pain management outcomes. Furthermore, our analysis indicates that reduced opioid consumption among ERAS patients aligns with the initiative's objective of utilizing multimodal analgesia, which emphasizes the use of non-opioid pain management strategies. This reduction is crucial not only for enhancing patient recovery experiences but also for addressing the broader public health challenge of opioid overuse. The contrasting results from the study by Dragun et al. (21), which did not examine opioid usage, indicate a gap in the literature regarding how ERAS protocols affect pain management. This underscores the need for future research to comprehensively evaluate opioid consumption and pain outcomes in ERAS studies, which could lead to more effective strategies for pain control in surgical patients. In summary, our findings contribute to the growing body of evidence supporting the implementation of ERAS protocols, particularly in enhancing recovery and reducing opioid reliance. Future studies should aim to standardize ERAS practices and examine the multifaceted impacts on patient outcomes across diverse surgical settings.

The analysis from our study indicated that the implementation of ERAS protocols significantly reduces overall hospital expenses, with the ERAS group incurring costs of 20,540 CNY compared to 23,789 CNY in the conventional care group (*p* < 0.001). These findings are consistent with existing literature, highlighting that ERAS pathways improve patient outcomes and lead to substantial cost savings (22). ERAS directly correlates with reduced hospitalization costs by minimizing the length of stay, facilitating early mobilization, and decreasing postsurgical complications. Additionally, a study (23) reported a mean cost saving of €1,022.78 per patient in the ERAS group, underscoring the financial benefits of these protocols alongside improved recovery outcomes. Our findings advocate for the broader adoption of ERAS across surgical disciplines, emphasizing its role as a strategic economic measure that benefits patient health and healthcare systems. Further research is needed to assess long-term outcomes and optimize ERAS implementation effectively.

### 4.1 The nursing role in ERAS for MISS

Nurses are essential in implementing ERAS protocols for minimally invasive spine surgery, significantly enhancing patient outcomes (24). Their role includes comprehensive preoperative education, holistic assessments, early mobilization, and effective pain management strategies. Collaboration with the multidisciplinary team (MDT) ensures cohesive ERAS implementation while nurses advocate for patient needs and preferences. By focusing on these critical areas, nurses enhance patient experiences and outcomes within ERAS frameworks, facilitating smoother recovery journeys for those undergoing minimally invasive spine surgery. Enhanced postoperative recovery necessitates the coordinated efforts of MDT members, particularly for patients with spinal trauma who often present with multiple comorbidities such as traumatic brain injury, diabetes, and heart disease. These patients benefit significantly from a multidisciplinary treatment approach that tailors effective care strategies to individual needs. During MDT sessions, nurses perform critical tasks, including organizing meetings, providing mental health support, and developing personalized nursing care plans. Research indicates that ERAS protocols, which emphasize a multidisciplinary approach, are particularly effective in improving accuracy and efficiency in patient care (25, 26).

### 4.2 Holistic nursing care

Individualized, holistic nursing care is crucial for patients undergoing spinal surgery. Studies show that such patients often experience significant surgical trauma, extended hospital stays, and heightened complication risks. Integrating ERAS protocols into spinal surgery workflows is essential, as nursing is pivotal in improving surgical outcomes (27). While postoperative complications are increasingly recognized, current pain management strategies require reevaluation. The Joint Commission (28) urges reducing opioid use for pain management due to risks like addiction, longer hospital stays, and higher costs. It promotes multimodal analgesia, prioritizing non-opioid therapies with opioids used when needed. This approach reflects evidence that non-opioid options are safer and more effective as healthcare providers face increasing accountability for the long-term impact of opioid prescribing.

### 4.3 Integrated nursing techniques

Recent findings suggest that while ERAS protocols can reduce hospital stays, they may inadvertently increase readmission risks (29, 30). Thus, successful ERAS implementation requires collaboration among all surgical team members and a focus on rehabilitation and nursing care extending beyond discharge (31). The primary goal of ERAS is to mitigate the surgical stress response, but patient engagement in their treatment is equally important. Establishing accessible follow-up clinics staffed with nurses and physical therapists is crucial for early rehabilitative care, including wound management, emotional support, and addressing muscle soreness.

### 4.4 Impact of nursing interventions on patient outcomes

The implementation of ERAS protocols by nurses significantly influences patient outcomes following MISS. Overcoming resistance to change requires collaboration between administrative and medical staff to streamline protocols and enhance patient pathways. E-health solutions facilitate quicker admissions and continuous monitoring, reducing LOS and costs without compromising care quality. Rehabilitation nursing interventions, including pre-and post-operative exercise education and psychological support, reduce pain levels and better prognostic outcomes than standard care. These measures promote early mobilization, shorten hospital stays, and lower the incidence of postoperative complications, highlighting the essential role of nursing in patient education and recovery. Nursing staff ensure that all patients are mobilized within the first eight hours of admission. For those unable to mobilize, prompt physical therapy evaluations are initiated. Early urinary catheter removal is advised by postoperative day one to prevent complications associated with prolonged immobilization. Adogwa et al. (32) found that early mobilization reduces perioperative complications and shortens hospital stays by 34% in the early ambulator cohort. Implementing nursing care protocols enhances patients' understanding of their conditions and promotes better pain management and daily activity performance.

### 4.5 Future perspectives

Future research should focus on long-term outcomes, comparative effectiveness of nursing strategies within ERAS protocols, and patient-centered outcomes like pain management and mobility. Integrating digital health tools for recovery tracking and communication will enhance care delivery. Investigating interdisciplinary collaboration's impact will provide insights into effective team dynamics. Additionally, exploring targeted psychological support for patients can address emotional distress, ultimately enhancing recovery and satisfaction. These directions will refine nursing roles and improve overall patient outcomes in MISS.

### 4.6 Study limitation

This study has several limitations. First, as a retrospective analysis, it lacked randomization and blinding, which may have introduced recall and selection biases. Second, it did not adequately account for confounding factors such as variations in surgical techniques and differences in nursing practices across institutions. These factors can significantly impact patient outcomes and may limit the generalizability of the findings. Variations in patient demographics, surgical methods, and nursing approaches may affect the applicability of the findings to broader clinical contexts. Future research should prioritize multicenter trials to include a more diverse patient population, employ randomized controlled designs to minimize biases, standardize protocols, and consider a broader range of confounding factors and long-term outcomes.

## 5 Conclusion

Implementing the ERAS protocol, supported by focused nursing interventions, significantly improved postoperative outcomes in patients with MISS. These improvements included shorter hospital stays, reduced opioid use, and minimized postoperative drainage. Additionally, the interventions helped prevent complications, readmissions, and reoperations. From an economic perspective, ERAS reduced hospital costs, demonstrating its cost-effectiveness. The findings underscore the crucial role of nursing care in optimizing clinical outcomes and healthcare efficiency. Future research should explore the implementation of ERAS in various settings to validate and further enhance these results.

## Data Availability

The original contributions presented in the study are included in the article/Supplementary Material, further inquiries can be directed to the corresponding author.
